# Hyperbaric Oxygen Therapy in the Treatment of Sudden Sensorineural Hearing Loss

**DOI:** 10.3390/jcm12041515

**Published:** 2023-02-14

**Authors:** George Psillas

**Affiliations:** 1st Otolaryngology Department, School of Medicine, Faculty of Health Sciences, Aristotle University of Thessaloniki, AHEPA Hospital, 54636 Thessaloniki, Greece; psill@otenet.gr; Tel.: +30-2310-994-762; Fax: +30-2310-994-916

Sudden sensorineural hearing loss (SSHL) is a frustrating and frightening experience for a patient. It occurs within 72 h and is defined as hearing loss of at least 30 dB at three or more consecutive frequencies. Several therapeutical methods have been applied so far for the treatment of SSHL. Steroids are the primary method, but vasodilators, anticoagulants, antioxidants, antivirals, vitamins and plasma expanders are also used [1]. 

The cochlea is an organ with a high oxygen demand. However, due to its location in the temporal bone, the blood supply is limited to a single terminal artery (labyrinthine). Therefore, the cochlea hair cells are vulnerable to a decrease in tissue oxygen supply [2]. According to the vascular etiologic theory of SSHL, the reduction in blood flow to the inner ear may result in local ischemia and there is an absolute need to increase the oxygen level in the perilymph. Studies have supported that the partial pressure of oxygen in the blood and via the capillaries of the cochlea increased significantly under hyperbaric conditions [3]. This process can minimize ischemic damage following SSHL and predispose a patient to vascular recovery [4]. Hyperbaric oxygen (HBO) was first used in the treatment of SSHL in the late 1970s [5].HBO can increase perilymph oxygenation when administered once daily in a multiplace hyperbaric chamber at a constant pressure of 2.2 atmospheres [6,7]; each HBOT session consists of two 40 min periods under 100% oxygen conditions, with an intervening 5 min air break to prevent oxygen toxicity [6]. 

According to the 2019 American Academy *Otolaryngology–Head and Neck Surgery* guidelines (updated) for the treatment of SSHL, HBO therapy remains an option but only when combined with steroid therapy for either initial or salvage therapy [8]. It was suggested that initial therapy should begin within 2 weeks of SSHL onset and that salvage therapy should start within 1 month of SSHL onset [8]. However, in the Consensus Conference on Hyperbaric Medicine in 2016, HBO combined with medical therapy was strongly recommended for use in patients with SSHL who presented within 2 weeks from disease onset [9].

In any case, the effectiveness of HBO is time-dependent, and effectivity decreases with increasing delay in administration [10]; Bayoumy et al. [11] recommended the initiation of HBO preferably within 24 or 48 h. Although several studies [12,13] support the notion that patients with severe or profound SSHL (mean hearing threshold at least 70 dB) were less likely to recover hearing after combined treatment with HBO and steroids, recent reviews and meta-analyses [14,15] have shown that adding HBO to steroid therapies might be of benefit in patients presenting with severe or profound SSHL.

There are limited studies comparing HBO to intratympanic steroids (combined with either oral or intravenous steroids) in the initial treatment of SSHL. Among those published, the work Hosokawa et al. [16] has demonstrated that the overall hearing recovery rate was significantly higher for the HBO + systemic steroids group than for the intratympanic + systemic steroid group (*p* < 0.001). For salvage therapy after failed systemic steroid treatment, a recent review [17] did not reveal a significant difference in hearing improvement between the HBO and intratympanic steroid group.

Based on the above findings and our experience, we suggest offering HBO combined with steroids (oral for mild degree of SSHL, intravenous ± intratympanic for moderate to profound SSHL) for patients with SSHL with any degree of hearing loss at presentation as soon as possible after SSHL onset (preferably within 7 days after onset).

In conclusion, this Special Issue gives new insights into the treatment modalities and the pathophysiologic mechanisms underlying SSHL. We invite submissions involving prognostic factors for SSHL treated with adjuvant HBO. Multicentric studies and collaboration are required to establish new therapeutical protocols and treatment options. Assessing the current knowledge and elucidating the effect of hypoxia on the cochlea blood supply will help us to promote our understanding of this complex clinical disorder.
